# New insights of platelet endocytosis and its implication for platelet function

**DOI:** 10.3389/fcvm.2023.1308170

**Published:** 2024-01-09

**Authors:** Yangfan Zhou, Jianzeng Dong, Mengyu Wang, Yangyang Liu

**Affiliations:** ^1^ Department of Cardiology, Cardiovascular Center, Henan Key Laboratory of Hereditary Cardiovascular Diseases, The First Affiliated Hospital of Zhengzhou University, Zhengzhou, Henan, China; ^2^ National Clinical Research Centre for Cardiovascular Diseases, Department of Cardiology, Beijing Anzhen Hospital, Capital Medical University, Beijing, China

**Keywords:** platelet, endocytosis, cargo contents, thrombosis, phagocytosis

## Abstract

Endocytosis constitutes a cellular process in which cells selectively encapsulate surface substances into endocytic vesicles, also known as endosomes, thereby modulating their interaction with the environment. Platelets, as pivotal hematologic elements, play a crucial role not only in regulating coagulation and thrombus formation but also in facilitating tumor invasion and metastasis. Functioning as critical components in the circulatory system, platelets can internalize various endosomal compartments, such as surface receptors, extracellular proteins, small molecules, and pathogens, from the extracellular environment through diverse endocytic pathways, including pinocytosis, phagocytosis, and receptor-mediated endocytosis. We summarize recent advancements in platelet endocytosis, encompassing the catalog of cargoes, regulatory mechanisms, and internal trafficking routes. Furthermore, we describe the influence of endocytosis on platelet regulatory functions and related physiological and pathological processes, aiming to offer foundational insights for future research into platelet endocytosis.

## 1 Introduction

Platelets are small, anuclear blood cells that derived from bone marrow megakaryocytes (MKs) or distant organs such as lung or liver, and play an essential role in hemostasis and thrombogenesis (1–3). At sites of vascular injury, platelets are instantly captured from blood stream, adhering to the subendothelial matrix at exposed endothelial surfaces to form thrombi (4). In addition to hemostatic function, platelets are instrumental in diverse physiological and pathological processes, including inflammation and immune response, antibacterial host defense, liver regeneration, tumorigenesis and metastasis (5). It has been proved that platelet activation is accompanied by the occurrence of exocytosis, during which platelet granules release their procoagulant contents to extracellular milieu. Furthermore, research on platelets has highlighted the critical role of endocytosis in their biological functions. Platelet endocytosis conception was first proposed more than 30 years ago, while the underlying mechanism of platelets endocytic pathways are relatively limited in definition (6).

Endocytosis usually refers to as the uptake of extracellular cargoes and the transport of cell surface receptor through various membrane-binding chambers named endosomes (7). With the plasma membrane invagination, extracellular substances are wrapped into cells and delivered to early endosomes. After that, early endosomes transmit the cargoes to distinct intracellular locations, such as degrading in the late endosomes, recycling to the platelet membrane, or secretion (8).

The phenomenon of platelet endocytosis has been acknowledged since the 1980s when the existence of active internal systems in platelets was first identified (7, 9, 10). Technological advancements have enabled the observation of platelets internalizing small particles (such as fibrinogen, IgG, and albumin) into membranous structures through a phagocytosis-like mechanism, as revealed by transmission electron microscopy (TEM) (11–15). Further research has unequivocally established the substantial role of endocytosis in platelet physiology. Beyond influencing the transport of granules and cargoes, platelet endocytosis is implicated in hemostasis and various pathophysiological processes (16). In this review, we aim to provide a concise summary of platelet endocytosis, covering the transported contents, molecular mechanisms, transport pathways, and its impact on platelet-associated physiological and pathological phenomena. This overview is intended to offer a systematic introduction and to foster novel insights for subsequent investigations into platelet endocytosis.

## 2 Cargoes uptake by platelets endocytosis

### 2.1 Plasma contents ingested by platelet granules

Platelets release more than 300 bioactive substances including multiple proteins, peptides and small molecules (nucleotides, serotonin, Ca^2+^, etc.), which mediate the diverse functions of platelets (17, 18). The bioactive substances are mainly packed in three kinds of intracellular secretory granules in platelet: α-granules, dense granules and lysosomes (19–21).

Platelet α-granules contain more than 280 proteins including coagulation factors, which are of fundamental importance in hemostasis (22). Some of these proteins are platelet-specific, for example, β3-thromboglobulin. Some counterparts are from plasma, including albumin, fibrinogen, fibronectin, von Willebrand factor (vWF), etc. Originally, it was believed that proteins in granules originated exclusively from *de novo* synthesis during megakaryocyte (MK) development. However, subsequent studies have revealed that circulating plasma proteins can be internalized into MKs or platelet granules via endocytic pathways, and then returned to the plasma (14, 23, 24).

Factor I (fibrinogen) is the primary adhesion protein secreted by platelets, accounting for 10% of α-granule proteins (12). Harrison provide the first clear evidence for *in vivo* endocytic uptake of fibrinogen from plasma by human platelet α-granules (23). Fibrinogen in platelets is not synthesized by MKs (25), and can only be obtained from plasma by endocytosis (12, 23, 26). It has been shown that factor III (Tissue Factor, TF) can be internalized by platelets into the open canalicular system (OCS) channels, followed by its accumulation in the platelet cytoplasm and, occasionally, within α-granules (24, 27, 28). However, the storage of TF in α-granule of unstimulated platelets is a subject of ongoing debate, since there is evidence of TF-mRNA in platelets (29). Factor V, necessary for the optimal conversion of prothrombin to thrombin, is present in significant concentrations in blood platelets and localized within the α-granules of unstimulated platelets. Research has localized factor V within the α-granules of unstimulated platelets (30, 31). Different from fibrinogen, factor V was endocytosed by MKs, but not by platelets (32). In addition to these coagulation factors, vascular endothelial growth factor (VEGF) (33), intravenously injected albumin, immunoglobulin G (14), insulin-like growth factor (IGF) I and IGF-binding protein-3 are also known to be ingested by MKs and incorporated into platelet α-granules (34), highlighting the complex and selective nature of protein uptake by platelets.

Dense granules within platelets contain small molecular substances, categorically divided into four primary types: 1. Nucleotides, including Adenosine Diphosphate (ADP) and Adenosine Triphosphate (ATP); 2. Amines, exemplified by serotonin; 3. Cations, such as calcium ions; and 4. Phosphates, notably pyrophosphate and polyphosphates (35). 5-hydroxytryptamine (5-HT), also known as serotonin, is a neurotransmitter. Platelets do not synthesize 5-hydroxytryptamine (5-HT), commonly known as serotonin, but rather acquire it from the bloodstream via the serotonin transporter (SERT), subsequently storing it in their dense granules (36). Serving as the primary carrier of serotonin in the blood, platelets play a pivotal role. The release of serotonin from platelets is crucial in enhancing the wound healing process in various organs (37).

### 2.2 Endocytic recycling surface receptors in platelets

A variety of receptors are expressed on the surface of platelets, enabling the recognition of extracellular ligands, matrix components, and receptors on other cells. Upon platelet activation, extracellular ligands bind to these surface receptors, providing a physical anchor and triggering the intracellular signaling events (38). Moreover, stimulation by agonist such as thrombin and ADP causes migration of platelet receptors from the interior to the cell surface and re-distribution (39–41). The dynamic turnover of endocytic and exocytic receptor trafficking contributes to cell invasion, metastasis and cytokinesis (42).

#### 2.2.1 Integrin αIIbβ3

Integrins are a family of adhesive receptors whose functional regulation depends not only on the conformational change but also via integrin trafficking (43). Recycling of integrins can rapidly deliver integrins back to the plasma membrane, thus providing the cell with a constant fresh reservoir for new adhesions (38).

Integrin αIIbβ3, uniquely expressed by platelets, serves as a key adhesion molecule pivotal in thrombosis control (44). Previous study has shown that there is a dynamic pool of αIIbβ3 in human platelets that is obviously internalized through the endocytic vesicles formation. Intriguingly, upon thrombin stimulation, these vesicles are capable of migrating to the cell surface (45). Consequently, the internalization process of integrin αIIbβ3 in platelets may constitute a regulatory mechanism for adhesion receptors (46). Huang et al. have elucidated the crucial role of αIIbβ3 endocytosis in platelets and proved that changes of integrin αIIbβ3 trafficking may influence platelet functions, particularly in terms of spreading and clot retraction (47).

#### 2.2.2 P2Y_1_ and P2Y_12_ purinergic receptors

ADP is of great significance for platelets activation. Two types of G protein-coupled receptors (GPCRs) expressed on platelet surface, P2Y_1_ and P2Y_12_, are activated by ADP (48, 49). Upon stimulation, P2Y_1_ is coupled to G_q_ and PLCβ, while P2Y_12_ is negatively coupled to adenylyl cyclase by G_i_) thus promoting complete platelet aggregation. ADP induced platelet activation is usually initiated by P2Y_1_ and amplified by P2Y_12_ receptor (50).

Mundell and colleagues have showed that both P2Y_1_ and P2Y_12_ could be internalized rapidly in human platelets by a radioligand binding approach (51). Their findings also revealed that inhibitors of protein kinase C (PKC) significantly diminish ADP-induced internalization of the P2Y_1_ receptor, while having no substantial effect on the P2Y_12_ receptor (51). With further research, they demonstrated that the endocytosis of P2Y_1_ and P2Y_12_ receptors relies on dynamin, which is indispensable for resensitization of responses (50). An intact putative postsynaptic density 95/disc large/zonula occludens-1 (PDZ)-binding motif is essential for proper internalization and subsequent recycling of the P2Y_12_ purinoceptor in human platelets (52). Moreover, The PDZ-binding protein Na^+^/H^+^ exchanger regulatory factor (NHERF) binds to the P2Y_12_ receptor to promote agonist-dependent internalization. In this process, arrestin can act as an adaptor to scaffold NHERF1 to these GPCRs thus motivating receptor internalization (53). Arf6 also participate in P2Y receptors internalization which will be discussed in the following text.

#### 2.2.3 CLEC-2

The hemi-immunoreceptor tyrosine-based activation motif–containing C-type lectinlike receptor 2 (CLEC-2) is a transmembrane protein that highly expressed in platelets and plays crucial roles in platelet activation (54). Besides contribution to thrombosis, platelet CLEC-2 is also involved in the regulation of tumor metastasis, blood-lymphatic vascular development, and inflammatory bleeding (55). CLEC2 regulates platelet activation mainly by influencing a Src- and spleen tyrosine kinase (Syk)-dependent tyrosine phosphorylation cascades.

Lorenz et al. have showed that by injecting INU1 (a monoclonal anti-body) *in vivo* can specifically remove CLEC-2 from platelets and MKs. Nonetheless, this intervention was observed to cause a marked decrease in CLEC-2 levels, leading to thrombocytopenia, which poses a challenge for its therapeutic use (55). The research indicated that the reduction in CLEC-2 due to INU1 is primarily a consequence of receptor internalization within the platelets, governed by the activity of Src-family kinases. Intriguingly, while the Syk kinase is not crucial for the decrease in CLEC-2 triggered by INU1, it is vital for the onset of thrombocytopenia. Thus, selectively inhibiting platelet Syk can counteract the thrombocytopenia caused by INU1, without interfering with the process of CLEC-2 internalization (55).

#### 2.2.4 GPIb

Platelet membrane glycoprotein (GP) Ib is a receptor for vWF. Binding of vWF to GPIb is important in the initiation of the process of platelet adhesion to damaged blood vessel walls (56). Previous studies have observed the presence of GPIb pools inside the platelets (57, 58). Redistribution of platelet surface and internal cisterna of GPIb was observed within platelets, during which endocytosis of GPIb receptor may occur (59). Jones et al. have established that platelet endothelial cell adhesion molecule-1 (PECAM-1) could regulate platelet GPIb internalization through dual AKT/protein kinase B/glycogen synthase kinase-3/dynamin-dependent and αIIbβ3-dependent pathways, so as to inhibit thrombin and vWF induced platelet activation (60).

#### 2.2.5 Mpl/TPOR

Thrombopoietin (TPO), together with its receptor, Mpl, can facilitate megakaryocyte differentiation, promote survival and proliferation of hematopoietic stem cells and progenitor cells (61). Receptor-mediated internalization appears to be the primary regulating way of plasma TPO level. Mpl could be internalized in human platelets, without recycling to the cell surface (62). Recent research suggested that endocytosis of Mpl was restricted in Dynamin 2 (DNM2) knock out platelets, which also resulted in phosphorylation of the tyrosine kinase JAK2 and increased TPO levels (63). In conclusion, in MKs, DNM2-dependent endocytosis plays important roles during its development.

### 2.3 Virus and bacteria

**Figure 1:**
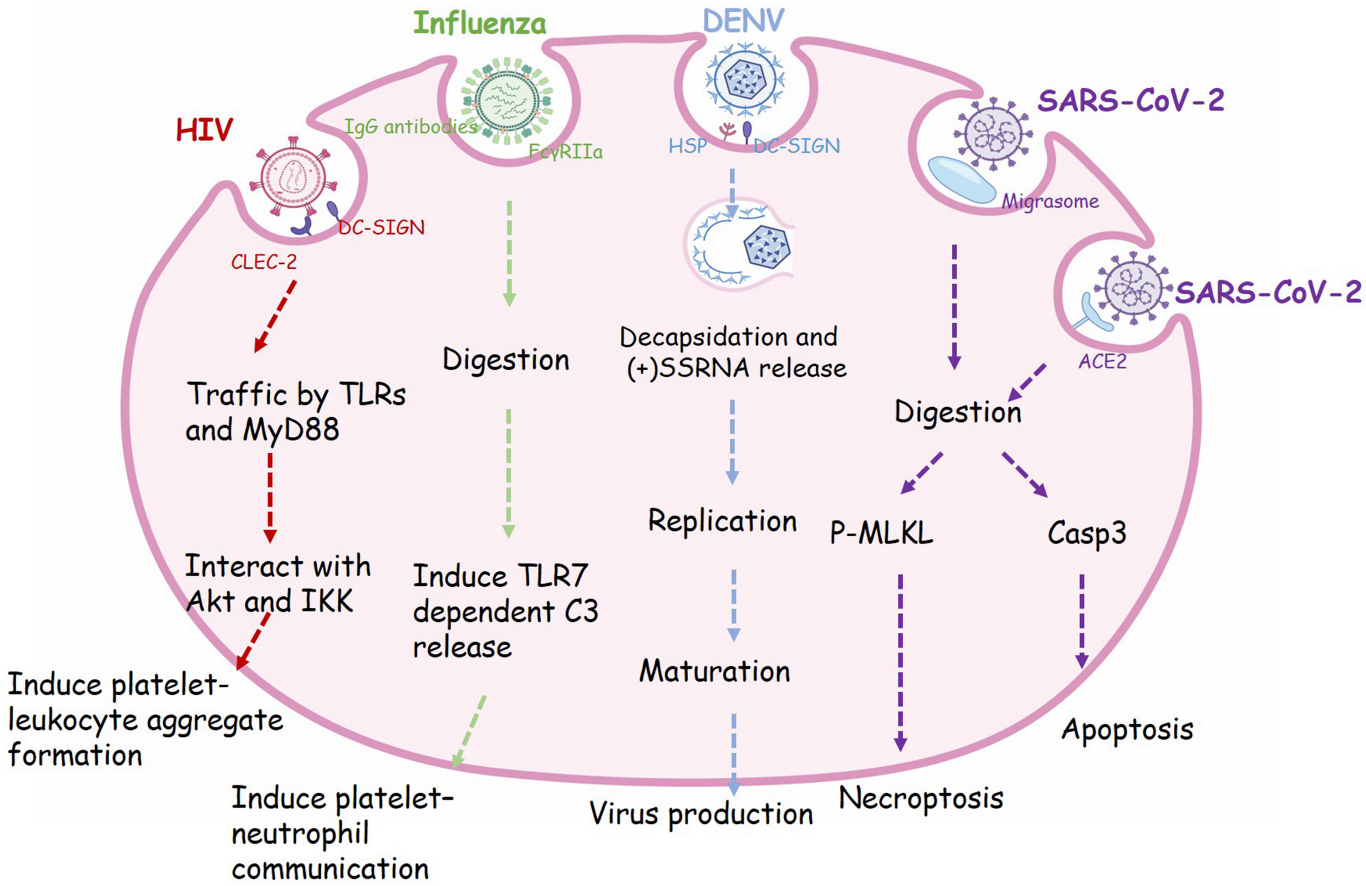
The phagocytic receptor, trafficking routes and regulatory functions of viral endocytosis in platelets. Many receptors expressed on the surface of platelets are involved in the process of virus phagocytosis. HIV enter platelets by binding to dendritic cell-specific ICAM-grabbing non-integrin (DC-SIGN) and C-type lectin-like receptor 2 (CLEC-2), then traffic via Toll like receptors (TLRs) and Myeloid Differentiation primary response protein 88 (MyD88) and interact with Akt and (IKK), finally induce granule release and platelet-leukocyte aggregate formation. Influenza viruses are recognized by IgG-specific antibodies and form an immune complex, which in turn is recognized by FcγRIIa, resulting in the internalization of this complex. Engulfment of influenza will activate platelet TLR7 and initiate granule release of complement C3 (C3), thus lead to NETosis. Dengue virus (DENV) binds to platelets via DC-SIGN and heparin sulfate proteoglycans (HSP). The viral particle go through decapsidation, ssRNA replication, maturation, and virus production. SARS-CoV-2 virions can be taken up into platelets through Angiotensin converting enzyme-2 (ACE2) or by attaching to microparticles. Phagocytosis of SARS-CoV-2 will induce apoptosis mediated by caspase 3 (Caps-3) and necroptosis mediated by phosphorylation of mixed lineage kinase domain-like pseudokinase (MLKL), finally lead to programmed cell death in platelets.

In addition to the hemostatic function, platelets also act as innate immune cells involved in inflammation and immune response *in vivo* (64). Platelets express some pathogen recognition receptors, e.g., complement receptors, αIIbβ3, FcγRIIa, CLEC-2, GPIbα and dendritic cell-specific intercellular adhesion molecule-3-grabbing non-integrin (DC-SIGN), which enable platelets to detect and interact with bacterial and viral particles (65). Several studies have shown that platelets can bind and phagocytose infectious microorganisms (66–69). Platelets could promote the transport of infection *in vivo* by taking up viruses, and they also may help the host organism to resist infection. It is proposed that herpes virus with larger DNA, such as herpes simplex virus (HSV)-1, only bind to platelets without internalization (70–72). In contrast, some smaller RNA viruses, are endocytosed by platelets, as shown in Figure 1 and described below.

#### 2.3.1 Human immunodeficiency virus type I (HIV1)

HIV1 is a single positive-stranded RNA virus belonging to the *Retroviridae* family, genus *Lentivirus*. In 1990, Zucker-Franklin D et al. first observed internalization of HIV1 and other retroviruses in MKs and platelets (73). In vitro experiments, Flaujac and colleagues incubated platelets with HIV viruses for 30 min. They found that HIV was completely internalized, maintaining the integrity of its ultrastructure. Characteristic endocytic vacuoles containing HIV particles were found near the plasma membrane (66). In addition, the endocytosis of HIV also occurred *in vivo* after test in platelets from clinical patients with AIDS (67). HIV-1 can be completely encased in platelets, transported through the body by circulating platelets and protected from attack by the host immune system (64). Platelets that internalize viruses undergo activation and exhibit P-selectin expression on their membrane. Subsequently, these P-selectin positive platelets are recognized by macrophages and efficiently cleared from the circulation (66). The thrombocytopenia complication after HIV infection may be partly caused by this defense mechanism (66).

#### 2.3.2 Influenza virus

Influenza is a respiratory virus, and infection of it will lead to barrier damage of epithelial-endothelial, allowing the entrance of virus into blood (68). In 1959, the incorporating of influenza virus into platelets was first observed by electron microscopy (74). Koupenova et al. proposed that incubation of platelets with H1N1 strain (WSN/33) leads to internalization of the viral particles by platelets, which is morphologically similar to phagocytosis, and the internalized virus are rapidly digested (75). As a matter of fact, almost all influenza A strains (H1N1, H5N1, and H3N2, etc.) can bind to platelets, albeit with varying affinities (H5N1 > H1N1 > H3N2) (76). Further studies are required to confirm whether and how these viruses are endocytosed into platelets. Overall, platelets may be a line of intravascular defense against influenza, as well as other immunity cells.

#### 2.3.3 Dengue virus (DENV)

Dengue fever is a mosquito-borne viral disease mainly found in tropical and subtropical regions. Infection of dengue virus in patients will cause capillary leakage, bleeding, and organ compromise, and in severe cases, it can lead to shock or even death (77). Platelets are crucial in the pathogenesis of DENV, as the infection is often accompanied by thrombocytopenia (78). Therefore absolute platelet count is used as lab indication to examine the disease progression of DENV (77). Researches have established that dengue induces platelet activation, mitochondrial dysfunction and apoptosis (79, 80). It is also suggest that DC-SIGN may participate as a key receptor in DENV mediated platelet activation (79). Ayo Y. Simon et al. found that DENV in blood can utilize DC-SIGN and heparan sulfate proteoglycan as primary receptors to bind to platelets directly. Platelets could endocytose this virus, replicate and produce infectious DENV. Usually platelets serve as an important line of defense against blood-borne viruses. However dengue appears to take advantage of this feature by getting inside the platelets and replicating further, increasing the survival chances of virus (78). The mechanism of DENV taking up by platelets remains unclear and requires further study, which can serve as new target for the DENV treatment.

#### 2.3.4 Severe acute respiratory syndrome coronavirus 2 (SARS-CoV-2)

SARS-CoV-2 is a new type of positive-sense single-stranded RNA beta coronavirus which induced coronavirus disease 2019 (COVID-19) pandemic (81). Infection of SARS-CoV-2 not only causes acute respiratory distress syndrome (ARDS), but also evokes a series of thrombotic complications, and leads to organ failure and even mortality (81, 82). These clinical complications of cardiovascular disease successfully raise concern about relationship between platelet activation and SARS-CoV-2.

Platelets in patients with COVID-19 exhibit hyper-activity, evidenced by elevated integrin αIIbβ3 activation and P-selectin expression (69). Observation of platelets from patients with severe SARS-CoV-2 infection showed viruses could enter platelets surface and intracellular platelets, suggesting that SARS-CoV-2 might be ingested by platelets. The mRNA traces of SARS-CoV-2 were detected in the isolated platelets, and virions could be observed by electron microscopy within platelets sections (69, 83, 84).

Milka Koupenova et al. found that the internalization and digestion of the SARS-CoV-2, changes in platelet morphology, and release of extracellular vesicles can be observed after co-incubating platelets with purified virions (85). Angiotensin converting enzyme-2 (ACE2) is recognized as the primary receptor for entry of SARS-CoV-2 into host cells (69). Koupenova and colleagues propose that both ACE2 dependent and independent endocytosis of SARS-CoV-2 occur in platelets. They also observed a class of microparticle related to platelets which might be migrasome (85). During cell migration, migrasome functions as local chemo-attractants and mediating release of cytoplasmic contents (86). Platelets can take up SARS-CoV-2 virions attached to microparticles (85). Following the internalization of SARS-CoV-2 by platelets, researchers have observed a colocalization of the virus with phospho-MLKL (mixed lineage kinase domain-like pseudokinase) and caspase 3. This interaction leads to a rapid onset of platelet cell death, during which the virus seems to fragment, inhibiting its further replication and spread. This suggests that platelets may play a protective role in the immune response against the virus. However, with the death of platelets, their internal active substances are also released, resulting in pro-thrombotic complications in COVID-19 patients.

#### 2.3.5 Bacteria

During bacterial infection and sepsis, direct and indirect interactions through a wide range of cellular mechanisms between platelets and bacteria may contribute to thrombocytopenia (87). In the microvasculature, bacterial cells could activate platelets, and cause thrombosis (88). Several studies have shown that *Staphylococcus aureus* (*S. aureus*) can induce aggregation and ATP release of platelets, and it is occasionally observed that *S. aureus* can be internalized by individual platelets (89–91). Moreover, platelet activation could increase the internalization of bacteria. During endocytosis, platelets appeared to elongate pseudopods that surrounded the *S. aureus* and completely enclosed the particles in a vacuole, in a manner similar to phagocytes engulfing bacteria. In the final stage of endocytosis, *S. aureus* seems to be closely contact with α-granule secretory fibrinogen within platelets (67).

In addition to *S. aureus* (a gram-positive bacterium), Porphyromonas gingivalis (*P. gingivalis*, a gram-negative bacterium) have also been reported to interact with platelets and cause aggregation of platelets (92). Xiangfeng Li et al. found that during bacterial induced platelet activation, *P. gingivalis* was engulfed in the OCS, and then internalized into the platelet cytoplasm. Streptococci and other bacteria have also been proved to interplay with platelets (88), however the detailed process of bacterial endocytosis into platelets has not been discussed and awaits further study.

### 2.4 Drugs endocytosis by platelet as smart drug delivery system

The controlled and efficient delivery system of drugs is essential for the clinical treatment of disease. An intelligent and controllable drug delivery system that effectively targets specific organs or tissues should maintain its bioavailability and minimize side effects (93). Platelets are endowed with the capacities of storage, transport, and release, making them potential carries for drug delivery systems (94). Moreover, when the drug is loaded in platelets, it could be protected from the clearance of immune system, and thus may allow a prolonged circulation time in blood.

Previous study has shown a strong link between platelets and cancer (95). Tumor cells can induce platelet adhesion, aggregation and release. During this process, the drugs loaded in platelets are released along with particulate components to the tumor site (96). The endocytosis properties of platelets can be used to load drugs, providing a promising way for guiding drugs to the blood circulatory system and complex tumor microenvironment.

#### 2.4.1 Doxorubicin

Doxorubicin (DOX) is a chemo-therapeutic drug used in the treatment of lymphoma, but it has many side effects such as short biological life time and cardiotoxicity due to non-specific bio-distribution (97). The utilization of DOX-loaded platelets as a drug delivery system, achieved by incubating doxorubicin (DOX) with a platelet suspension at 37°C, has been investigated in multiple studies, particularly focusing on lymphoma treatment (93, 96). By this means, the researchers successfully prolonged DOX retention time compared to synthetic drug delivery systems (96, 98). Dox-loaded platelets facilitate the gathering of intracellular drug through platelet aggregation caused by tumor cells and release DOX in a pH-controlled manner. This method could reduce the side reaction of chemotherapy drugs to the patient and improve the therapeutic effect in clinical treatment of lymphoma (99). However, the precise mechanism underlying platelets' internalization of doxorubicin (DOX) remains unclear, as it is not explicitly detailed in these studies. The process of DOX encapsulation within platelets likely involves intricate biochemical pathways, possibly engaging the open canalicular system (OCS) of the platelets (96, 98). This area warrants further investigation to elucidate the exact mechanisms involved.

#### 2.4.2 Monoclonal antibody bevacizumab

Platelets are the main source of VEGF in blood. Platelets activation leads to release of growth factors from α-granule, including VEGF, which promotes angiogenesis (100). Bevacizumab is a monoclonal antibody that blocks VEGF and has been used in the treatment of cancer disease. Previous study demonstrated that platelets can take up bevacizumab (101). Immunofluorescence microscopy displayed that bevacizumab phagocytosed in platelets colocalized with P-selectin. They found that platelets from patients receiving bevacizumab treatment can endocytose bevacizumab (101). Platelet endocytosis is still worthy of further study in the direction of drug delivery with platelets as carriers, which is of great significance for targeted treatment of tumors and cardiovascular diseases.

## 3 Regulators of platelet endocytic machinery

**Figure 2:**
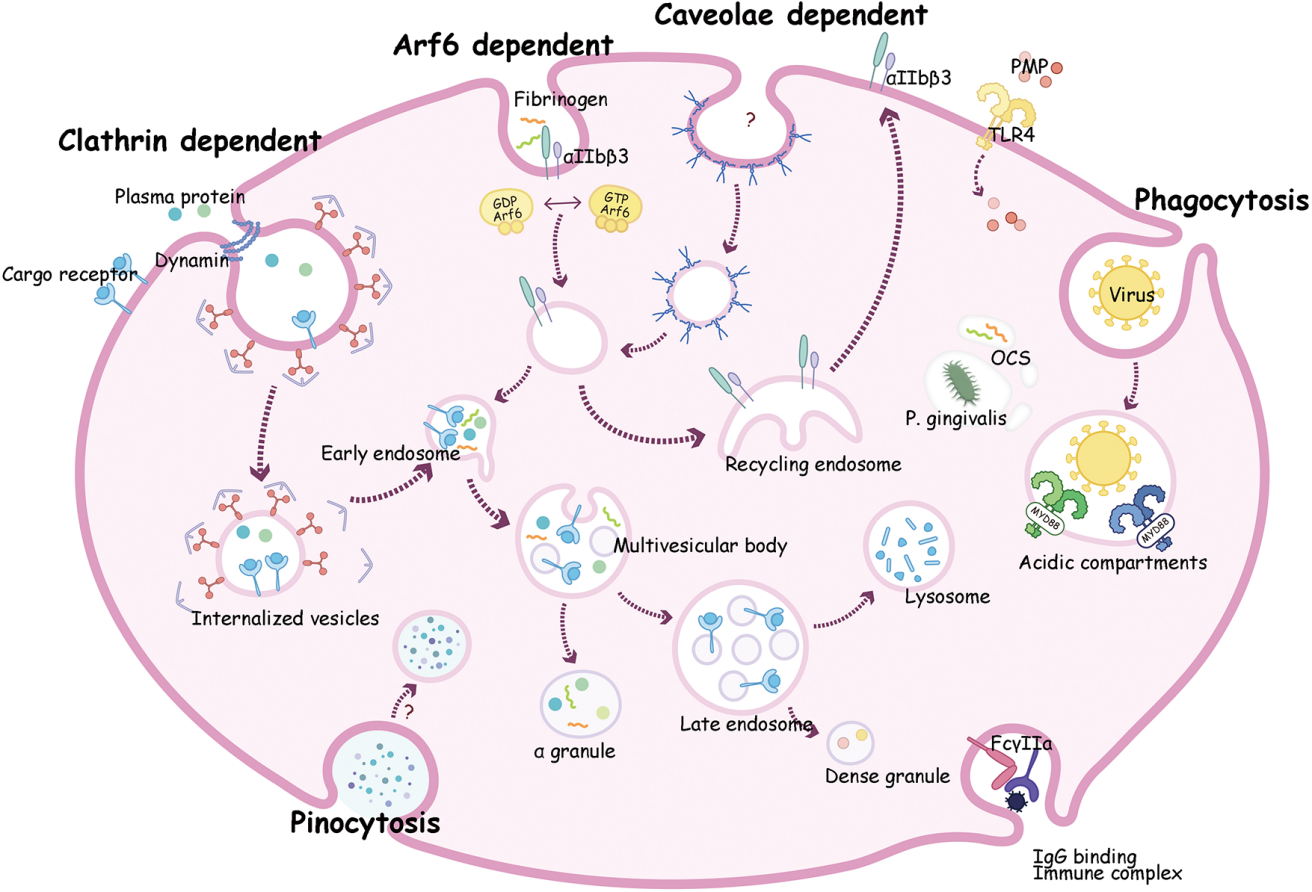
A composite description for endocytosis mechanism and transportation pathways in platelets. Clathrin dependent endocytosis can transport plasma proteins such as vWF and fibrinogen (Fg) with or without receptor into platelets. These proteins go through early endosome then be sorted to multivesicular bodies (MVB) and ultimately into α-granules for storage. Alternatively, cargoes can move into late endosomes then transit into dense granules or to lysosomes where it may be degraded or stored. Receptors such as P2Y_12_ and αIIbβ3 are taken up by Arf6, then circulate to recycling endosome, and finally return to the plasma membrane. Virions may enter platelets by phagocytosis, then bind to endosomal TLRs, and activate a myeloid differentiation primary response protein 88 (MyD88)- based signaling cascade, and finally been digested. Proteins like fibrinogen and bacteria such as *P. gingivalis* can be internalized into the open canalicular system (OCS) channels.

Platelet endocytosis has been long recognized, however, the molecular machinery, remain understudied. Up to now, several molecules have been proved to be involved in the regulation of platelet endocytic mechanism (Figure 2). According to existing studies, multiple pathways of endocytosis could be broadly classified as clathrin-dependent and clathrin-independent internalization (102).

### 3.1 Clathrin dependent endocytosis

Clathrin is a protein which can form a polymer on the downside of the coated pit. Endocytosis mediated by clathrin usually depends on clathrin-coated vesicles (103). In platelets, clathrin-coated membranes were first noticed in the 1980s (9). The formation of clathrin encapsulated vesicles requires the following five steps: initial stage, selection of transport goods, coat assembly, scission and coating removal (103). In this process, extracellular molecules integrate with the ectodomain of receptors, then accumulate in coated pits on the cell membrane (16). The pits are further transformed into clathrin-coated vesicles with short-life (104). Subsequently, the coat of vesicles falls off, and the remaining portion fuses with endosomes and is transport along the endocytosis pathway (42, 105). By utilizing a novel inhibitor of clathrin named pitstop 2, Wen Gao et al. suggest that integrin αIIbβ3 trafficking mediated by clathrin controls spreading during platelet activation (42).

#### 3.1.1 Dynamin

Dynamins (DNMs) are highly conserved mechanochemical GTPases regulate endocytosis and vesicle transport (63, 106). After the formation of clathrin-coated vesicles, DNM is recruited to promote the vesicle scission from the neck (42). There are three members of DNM family: DNM1, DNM2, and DNM3. Human platelets express all DNMs, whereas mouse platelets mainly express DNM2 (107, 108).

Platelets contain low levels DNM1, as it mainly expressed in brain (109). Previous studies have suggested DNM3 participate in MK maturation and platelet formation (110, 111). DNM2 mutations are associated with thrombocytopenia and hematopoietic diseases (112, 113). Bender et al. found that deletion of DNM2 in platelets significantly suppressed the endocytosis of the TPO receptor, Mpl. In conclusion, DNM2-dependent endocytosis is essential for megakaryopoiesis, thrombopoiesis, and bone marrow homeostasis (63).

#### 3.1.2 Disabled-2 (Dab2)

Disabled-2 (Dab2) acts as an adaptor protein in clathrin-mediated endocytosis, and participates in transport of many receptors and intracellular signaling (114). Dab2 has two alternative splicing isoforms p82 and p59 (115). P82-Dab2 is mainly distributed in the cytosol and on α-granules of human platelets, which regulates fibrinogen binding and platelet aggregation (116). P59-Dab2 is abundant in mouse platelets, and it is essential for fibrinogen ingestion, RhoA-ROCK activation, ATP secretion, and integrin αIIbβ3-mediated signaling (117). Knock out of Dab2 specifically in mice platelets showed a bleeding tendency and impaired thrombosis. It has been proved that Dab2 shows strong interaction with Gα₁₂/₁₃-mediated thrombin signaling in hemostasis (118). Dab2 is a key player in the endocytosis of platelet Integrin-β3. Cheng-han Yu and colleagues have reported that the turnover of Integrin-β3 in platelets is modulated in a manner dependent on cell-matrix force interactions. Under the influence of traction forces, talin directly associates with Integrin-β3, leading to the formation and maturation of focal adhesions. Conversely, the absence of physical forces on RGD-glass triggers the recruitment of Dab2 and clathrin, culminating in the internalization of activated integrins (119).

#### 3.1.3 IFN-induced transmembrane protein 3 (IFITM3)

IFITM3, an type I interferon (IFN)-responsive gene that plays critical roles in restriction of viral replication (120). During infection of dengue and SARS-CoV-2 virus, expression of IFITM3 significantly increased in MKs and platelets, thus limiting the entry and replication of virus in the cytoplasm of MKs (121). IFITM3 has been shown to interact with clathrin and αIIb and change their plasma membrane localization into lipid rafts, thereby mediating endocytic transport process in platelets (122). After administration of IFN in mice, endocytosis of fibrinogen and platelets reactivity significantly increased in an IFITM3-dependent manner. In nonviral sepsis, platelet IFITM3 expression elevated, leading to an increase fibrinogen content in platelets and thrombosis. These data support IFITM3 as a regulator of clathrin-dependent endocytosis in platelets, hyperreactivity, and thrombosis during inflammatory stress (122).

### 3.2 Clathrin independent endocytosis

The most common non clathrin-coated pits are known as caveolae (123). Caveolae-mediated endocytosis forms flask-shaped invaginations decorated with caveolin proteins on the surface of plasma membrane (124, 125). There are three types of caveolin proteins in mammals: caveolin1, caveolin 2, caveolin 3. Caveolin 3 is muscle specific, while the other two are widely expressed in non-muscle cells (123). Human platelets contain all three types of caveolin; however, whether and how these proteins coupled to endocytosis in platelets is still unknown (126).

RhoA and Cdc42 (small G proteins from the Rho family) have been widely involved in clathrin-independent endocytic regulation and the control of cytoskeletal recombination and intracellular signaling events (123). RhoA recruits the actin machinery to produce membrane invaginations in the process of endocytosis (16). Deficiency of RhoA not only significantly inhibits platelet aggregation, granule secretion, spreading on fibrinogen and other coagulation functions, but also induces macrothrombocytopenia (127). Meanwhile, Cdc42 plays crucial roles in platelet aggregation in response to collagen and integrin α2β1 activity (128). However, whether they showed priority for platelet endocytosis contents remains unclear and required further investigation.

#### 3.2.1 The Arf GTPase family

The Ras-like, small guanosine triphosphate (GTP)–binding proteins named adenosine 5′-diphosphate–ribosylation factors (Arf's), play essential roles in intracellular trafficking (47). Based on sequence homology, they are separated into three types largely as Class I (Arfs 1–3), Class II (Arfs 4–5) and Class III (Arf6) (6). Among them, Arf6 has been most concerned in platelets. In rest platelets, Arf6 exists in an active, GTP-bound state. Upon stimulation by collagen and convulxin, the level of Arf6 in platelets rapidly decreased, by converting it to an inactive Arf6-GDP form (129). This process is regulated by the primary signaling activated by PAR receptor and GPVI, or contact-dependent signaling by integrin αIIbβ3 (130). When switch between active and inactive state, Arf6 facilitates internalization of ligand and recycling of receptors (131). In human platelets, Arf6 regulates the internalization and function of P2Y_1_ and P2Y_12_ purinoceptors. Moreover, Arf6 can regulate Nm23-H1 activation, a nucleoside diphosphate kinase, then facilitate fission of coated vesicles in a dynamin-dependent manner during endocytosis (132). Huang et al. generated mice with Arf6 deficiency in platelets, they found that hemostasis phenotypes (e.g., aggregation, ADP secretion, tail bleeding times, and occlusion time of arterial thrombosis) were normal, yet, Arf6 deficient platelets displayed enhanced spreading on fibrinogen and accelerated clot retraction. It is worth noting that fibrinogen uptake and storage were defective in Arf6^−/−^ platelets (47). Thus, Arf6 may selectively regulate endocytic trafficking of platelet αIIbβ3 and the efficacy of platelet function. Arf1 and Arf3 are more abundant than Arf6 in platelets (6). In addition, several Arf regulators such as the GAPs, GEFs, ASAP1/2, ArfGAP1/2, and ARAP1 are present in platelets (107). Their regulatory roles in platelet endocytosis required further investigations.

#### 3.2.2 Toll like receptor-4

Platelets can remove pathogens quickly through toll-like receptors (TLRs), and their endocytic and phagocytic abilities proved their association with the innate immune mechanisms (133). A variety of TLRs are expressed in platelets, some of which are expressed on platelet membranes and some of which are expressed inside platelets. Previous study revealed that platelets internalized circulating platelet microparticles (PMP) through TLR-4 and TLR-4 related components (133). It has been confirmed that PMP internalization in platelets could proceeds thrombus formation. Thus blockade of TLR-4 mediated endocytosis might be a potential target for antithrombotic therapies in pathological situations.

### 3.3 Phagocytosis and pinocytosis

In certain cells, extracellular substances waiting to be internalized are gradually surrounded by the invaginations forming on the plasma membrane, and then taken up into cells. This process is called phagocytosis (123). Phagocytosis usually refers to engulfment of large particles such as dead cells or invading microbes (16). Bacteria and viruses seem to be ingested by platelets in a process akin to true phagocytosis (67, 68, 134). Platelets express some pathogen recognition receptors, such as FcγRIIa, αIIbβ3, GPIbα, CLEC-2, and DC-SIGN, which can sense bacterial and viral particles and mediate phagocytosis of platelets (65). FcγRIIa is indispensable for IgG-containing complex clearance. Platelets can phagocytose immune complexes from circulation in a FcγRIIa-dependent manner (135, 136). It has been proved that during viral infection, influenza viruses are recognized by IgG-specific antibodies and form an immune complex, which in turn is recognized by FcγRIIa, resulting in the internalization of this complex, and activating platelets (68). Besides FcγRIIa, DC-SIGN, mainly expressed in macrophages and dendritic cells, also gets involved in in phagocytosis of platelets (66). As mentioned before, DENV directly bind to platelets utilizing DC-SIGN and heparan sulfate proteoglycan as primary receptors, and mediating entry of DENV into platelets (78, 137). Moreover, previous findings support that DC-SIGN can modulate HIV phagocytosis in platelets with CLEC-2 acting as its partner (138). In conclusion, these lectins are required for efficient binding of HIV and platelets, and might play important roles in phagocytosis of virus into platelets (Figure 1).

Different from phagocytosis, pinocytosis is another form of endocytosis that small particles are taken into the cell suspended within small vesicles. Pinocytosis usually participates in the absorption of extracellular fluids. It has been reported that fibrinogen might be taken up into platelets by pinocytosis, but its specific regulatory mechanisms and influencing factors need further research (31).

## 4 Endocytic trafficking routes in platelets

Platelets take up different cargoes, but what happens next? After particles enter platelets, they will go through different transport pathways, and exerting their respective regulatory functions.

Platelets contain distinct membrane-bound compartments (endosomes) that exercise the function of “transfer stations” for internalized cargo molecules (47). Platelets have a weak ability to synthesize proteins, so most cargo is taken from plasma. OCS is a complex a system of tunneling invaginations of the plasma membrane found in platelets that connects with membrane channels and cell surfaces to control the endocytosis and exocytosis (139, 140). The role of the OCS in the regulation of platelet functions can be outlined as follows: 1. The OCS is instrumental in the endocytosis of plasma proteins and other circulating molecules for example fibrinogen and TF (23, 24). This process allows platelets to acquire components that they do not synthesize themselves. 2. The OCS aids in the internalization of bacteria such as *P. gingivalis* and viral pathogens such as HIV (67, 92). This interaction is crucial for the immune functions of platelets, contributing to their role in inflammation and defense against infections (141). 3. The potential of the OCS in clinical treatments, particularly as a drug delivery system, is highlighted by its role in the encapsulation of drugs like DOX in platelets, as demonstrated in previous studies (96). This suggests that manipulating the OCS structure could be a novel approach in drug delivery applications. It has been observed that a distinct closed canalicular system of dispersed membrane-bound structures also exist in platelets (142). During early platelets activation, the closed canalicular system fuses with the plasma membrane and becomes open, thus these closed canalicular systems might represent endosomes in platelets (16, 142).

Platelets can sort endocytosed cargoes into different endosomal compartments (Figure 2). To analyze the sorting routes in platelets, researchers use anti-Rab4 (represent early endosomes), anti-Rab11 (recycling endosomes), and anti-Rab7 (late endosomes) antibodies as markers of different compartments. Both Rab4 and Rab11 compartments were present in platelets, but not overlapped, indicating that resting platelets contained both early and recycling endosomes (7, 8, 129). The endocytosis trafficking in platelet is time-dependent. As shown in Figure 2, internalized cargo transports through early endosomes, where it can be selected to recycling endosomes, then return back to the plasma membrane (e.g., αIIbβ3, P2Y_12_, TPOR), or to multivesicular bodies (MVB) and finally enter α-granules for storage (e.g., fibrinogen, vWF, thrombospondin-1). Either directly from early endosomes or through MVB, cargoes can also transit into late endosomes, and ultimately move into dense granules or lysosomes for storage or degradation (143, 144).

In the process of platelet endocytosis transport, a series of proteins are required to facilitate membrane fusion. Soluble *N*-ethylmaleimide-sensitive factor attachment protein receptor (SNARE) can mediate inter-compartmental transport in most cells, but its function in platelets is still under investigation. Cellubrevin/Vesicle-Associated Membrane Protein-3 (VAMP-3) is a v-SNARE protein localized to punctate structures within platelets. It has been reported that VAMP-3 not only regulate membrane, but also facilitate platelet aggregation and secretion (145, 146). Banerjee and colleagues have described the role of VAMP-3 in mediating endocytosis and endosomal trafficking in platelets, as well as controlling intracellular uptake/accumulation of fibrinogen and transferrin, platelet spreading, clot retraction, and proper regulation of TPOR signaling (7).

As mentioned before, TLR4 is involved in platelet endocytosis of extracellular pathogens. After the internalization of pathogens such as bacteria and viruses, platelets may also interact with other TLRs of endosomes. For example, TLR7 in the endosome recognizes single-stranded RNA and can be activated by retroviruses (147–149). Platelet TLR9 is detected both on the platelet membrane and in the endosome (150). HIV-1 pseudovirions incubation could induce endocytosis and trafficking of virions to an acidic, degradative compartment in platelets. Subsequently, platelets were activated with granule release, and platelet-leukocyte aggregate (PLA) formation through the pathway involved in activation of TLR7/TLR9 and myeloid differentiation primary response protein 88 (MyD88), as well as the participation of downstream interleukin 1 receptor associated kinase 4 (IRAK4), Akt, and I*κ*B kinase (IKK) cascade signaling (65).

## 5 Perspectives and conclusion

In contrast with karyocytes, platelets are anucleated but relatively complex cells, with extensive endomembrane systems, and more hectic intracellular transport activities than ever thought. Endocytosis process has great impact on the up take, storage, and activity of proteins in platelet granules. This may have significant implications in clinical treatment of human diseases, as alterations in platelet granule proteins may contribute to thrombosis or bleeding. Endocytosis is a multi-step process that utilizes several routes of cargo entry/transit/exit (16), it also involved in various functions of platelets. Endocytosis might permit platelets to take up several prothrombotic and proinflammatory mediators (e.g., fibrinogen, vWF, IgG) from plasma; these messengers can then be stored into α-granules and later released during activation. Platelets endocytosis mediates surface receptors recycling: for example, integrin αIIbβ3 trafficking may affect platelet spreading and clot retraction, and internalization of both P2Y_1_ and P2Y_12_ receptors is required for resensitization of platelet responses (50). In addition, TPO receptor Mpl endocytosed by MK/platelets could regulate megakaryopoiesis, thrombopoiesis, and bone marrow homeostasis (63). Endocytosis may also enable platelets to function as immune cells since it can phagocyte bacteria and virus. Moreover, endocytic contents from the extracellular environment may provide platelets with the ability to differentially respond to corresponding physiological and pathological stimuli, thus modulating platelet interactions with other cells (16).

From the perspective of clinical disease treatment, platelet endocytosis may also play important roles. For example, in patients with nonviral sepsis, the expression of IFITM3 and contents of fibrinogen significantly increase in platelets, indicating that endocytosis mediated by IFITM3 may affect platelets reactivity and thrombosis during inflammatory stress (122). A recent report has suggested that endosomes play a crucial role in platelet activation, particularly in disease-specific contexts such as immune-mediated inflammatory diseases (151). Hydroxychloroquine (HCQ), the mainstay treatment for systemic lupus erythematosus, has been reported to function through limiting acidification of endosome in platelets (152). This underscores the considerable potential of platelet endosome research in revolutionizing diagnostics and therapeutic strategies across a spectrum of clinical disciplines. Additionally, a deeper understanding of platelet endocytosis and its integral role in platelet biogenesis is essential, not only for the validation of ex vivo platelet production for transfusion purposes but also for pioneering applications in the realms of inflammation, infection, and oncology. Circulating platelets, as temporally-regulated reservoirs, are replete with vital constituents sourced from the bone marrow and finely tuned by the circulatory environment. Looking ahead, we envision platelets, with their unique endocytic, transport, and release capabilities, as formidable candidates for the development of advanced, targeted drug delivery systems, especially in the context of clinical oncology.

In this review, we have encapsulated the latest research on platelet endocytosis, delving into the intricacies of endosome contents, the key regulatory elements, and the complex pathways of delivery. Research in the field of platelet endocytosis is still nascent, with numerous aspects of this process yet to be unraveled. As technological advancements continue to emerge, we anticipate a deeper and more comprehensive exploration of this domain. We hope that this review will provide researchers with a comprehensive insight into the intricacies of platelet endocytosis and transport, paving the way for further discoveries.
